# Identification of birch pollen species using FTIR spectroscopy

**DOI:** 10.1007/s10453-018-9528-4

**Published:** 2018-07-04

**Authors:** Joanna Depciuch, Idalia Kasprzyk, Elzbieta Drzymała, Magdalena Parlinska-Wojtan

**Affiliations:** 1 Institute of Nuclear Physics Polish Academy of Sciences, 31342 Kraków, Poland; 2 Department of Ecology and Environmental Biology, University of Rzeszow, Zelwerowicza 4, 35-601 Rzeszow, Poland

**Keywords:** *Betula*, Birch, FTIR spectroscopy, Pollen, Palynotaxonomy, SEM

## Abstract

In this study, the morphology and chemical composition of pollen grains of six birch species (*Betula utilis Doorenbos*, *B. dahurica*, *B. maximowicziana, B. pendula*, *B. pubescens* and *B. humilis*) were examined to verify which of these features allow distinguishing them in a more unambiguous way. For this purpose, scanning electron microscopy and light microscopy, as well as Fourier transform infrared (FTIR) spectroscopy and curve-fitting analysis of amide I profile, were performed. The microscopy images show that the pollen grains of *B. pubescens*, *B. pendula* and *B. humilis* are similar in diameter and significantly smaller than those of others species, with the largest diameter observed for *B. utilis Doorenbos*. However, the results obtained from FTIR spectroscopy indicate that the chemical compositions of *B. pubescens* and *B. pendula* are similar, but *B. humilis* is outlaying. Summarizing, it is not possible to unambiguously state, which feature or which technique is the best for differentiating between the six chosen birch species. However, the study showed that both techniques have potential for identification of birch pollen species.

## Introduction

Modern plant taxonomy is based on anatomical and morphological, as well as on genetic, biochemical and physiological studies (APG II 2003). One of many features, which could be used in taxonomy, is the morphology of pollen grains. Pollen grains have a very large variety of forms. The most important taxonomic parameter is the surface sculpture of the cell wall termed exine. The other indicative features are shape, grain size, as well as number, type and position of the apertures (Erdtman 1952). The pollen grains are identified with different accuracies. Often, it is not possible to identify pollen grains to species level, as in the case of *Betula*, *Alnus*, *Populus* or *Artemisia.* For some species, it is possible to identify pollen grains only to higher taxonomic categories like family (Poaceae) and even type—Plantago major/media (Frenguelli and Kasprzyk 2015).

Attempts to distinguish *Betula* pollen grains to the level of species were undertaken by Birks (1968), Mäkelä (1996) and Caseldine (2001). These authors, using the biometric method, indicated that the smallest pollen grains are *Betula nana* (L.), followed by *B. pendula* (Roth.) and *B. pubescens* (Ehrh). They showed that although the diameter of pollen grains of some *Betula* species differs noticeably, the frequency distribution curves of the pollen sizes overlap; thus, it is difficult to reliably identify grains to species level. Birks (1968) indicated the diameter/pore depth ratio as good criterion to distinguish between *B. pubescens* and *B. nana* grains. Clegg et al. (2005) gave the threshold size for pore depth and diameter/pore depth ratio to distinguish pollen of tree from shrub birch species native for western North America. Blackmore et al. (2003) presented the key to determine the *Betula* pollen types such as *B. pubescens* type comprising *B. pendula*, *B. pubescens* ssp. *carpatica*, *B. pubescens* ssp. *pubescens*, *B. pubescens* ssp. *tortuosa* and *B. nana* type including *B. nana* and *B. humilis* (Schrenk). These types were distinguished based on the size and shape of grains, the sculpture of exine, as well as the structure of pores, especially the vestibulum.

Pollen analysis is still based on light microscopic observations. This method requires enormous experience and is additionally time-consuming. That is why more and more scientists seek for other solutions without losing the quality of identification and simultaneously simplifying the work. An alternative to microscopic analysis could be the identification of genetic material using techniques of molecular detection (Zhou et al. 2007; Parducci and Suyama 2011). Furthermore, pyrolysis mass spectrometry (PyMS) can also discriminate plants phylogenetically (Kim et al. 2004a, b). However, biomarkers determined by PyMS data cannot be deconvoluted to identify the chemical compounds (Kim et al. 2004a, b). This problem can be solved by Fourier transform infra red (FTIR) spectroscopy. Indeed, the pollen grains of some species are morphologically similar, but differ significantly in chemical composition; thus, these differences can be revealed by spectroscopic methods and visualized in the obtained spectrum. Pappas et al. (2003) were the first to demonstrate that infrared spectroscopy can be used for the determination of pollen grains to the species level, creating libraries of pollen spectra. Furthermore, they compared the results obtained from FTIR with light microscopy observations. Moreover, FTIR enables chemical analysis of pollen samples for plant phenotyping to study plant–environment interactions, such as influence of climate change, stress or pathogens (Bağcıoğlu et al. 2017; Zimmermann et al. 2017; Lahlali et al. 2014). Furthermore, other authors used FTIR spectroscopy to determine the connection between the viability and the germination capacity (Buta et al. 2015). The attempts of discrimination and classification of allergy-relevant pollen using FTIR spectroscopy combined with multivariate statistical methods were made (Dell’Anna et al. 2009). In this work, the authors confirmed that the hierarchical cluster analysis provided valuable information about the reproducibility of FTIR spectra of the same taxon. The utility of infrared spectroscopy in palynology was demonstrated by Gottardini et al. (2007). They showed that it is possible to effectively apply this method for the determination of pollen grains to the species level. Moreover, using FTIR spectroscopy it was possible to identify grass pollen grains to subfamily level with an 80% success rate (Julier et al. 2016). This works are very important, because uniform morphology of different species of *Poaceae* (grass) pollen means that identification to below family level using light microscopy is extremely challenging. Large-scale studies on the identification potential of the pollen grains by means of infrared spectroscopy were initiated by Zimmerman and colleagues (2014). They successfully applied this technique to identify the pollen grains of coniferous plants, showing that pollen grains of closely related species with similar morphology distinctly differ in chemical composition (Zimmerman 2010). In their subsequent studies, they prepared spectra libraries of 300 different species belonging to 53 angiosperms families (Zimermann and Kohler 2014; Zimmermann et al. 2015, 2017).

FTIR spectra of individual pollen samples are different, and the most visible differences are in the spectral regions corresponding to vibrations originating from lipids, sporopollenin and carbohydrate. Spectral variability enables sufficient differentiation of plant-related families and genera (Zimermann and Kohler 2014, 2015), and even congeneric species (Bağcıoğlu et al. 2015; Zimmermann 2018). Furthermore, FTIR has been used in plant biology to differentiate between cell wall mutant plants (Stewart et al. 1997; Chen et al. 1997). Those studies showed that FTIR could be used to identify structural and architectural alterations in cell walls.

The purpose of the investigation reported here is to compare the morphology and chemical composition of pollen grains of chosen birch species. For this purpose, infrared spectroscopy (FTIR), curve-fitting analysis of amide I profile, light and scanning electron microscopy (SEM) were applied. This study will allow determining whether morphological or chemical features are better for the distinction between birch species.

## Materials and methods

### Pollen samples

The study was carried out in Bolestraszyce Arboretum, SE Poland. This arboretum (22°50′E; 49°54′N) covers an area of nearly 29 ha. Given the undulating topography, the altitude ranges between 195 and 216 m a.s.l. The climate of the Bolestraszyce region is classified as warm temperate. The mean annual total precipitation and mean annual temperature are 600 mm and 8.2 °C, respectively. The arboretum collection comprises 2200 taxa of trees and shrubs including native species, fruit trees as well as plants of foreign origin. For the present experiment, six birch species were chosen: *B. pendula* (Roth.), *B. pubescens* (Ehrh.), *B. humilis* (Shrank), *B. maximowicziana* (Regel), *B. utilis* (D. Don) *Doorenbos* and *B. dahurica* (Pall.). These species differ in origin, biology and environmental requirements. The first three species are native to the Polish flora; the remaining, non-native, are planted in urban greenery, home gardens and do not occur in natural habitats. They are generally fast-growing, prefer light and tolerate dry, infertile soils. *B. pendula* is an important forest-forming species and is thus considered as key woodland pioneer species, growing well on infertile soils. The rarely occurring in Poland *B. pubescens* prefers fertile, fresh, wet and even swampy soils, which also prefers *B. humilis*. Pollen of each species was collected from several inflorescences in April 2016 during flowering phenophase. If the individuals pollinated abundantly, pollen was collected into an Eppendorf tube in situ. In other cases, the branches with inflorescences were taken to the laboratory and were put into containers with water for several days until the inflorescences started to release pollen. Collecting material from all species in the same location greatly reduces the influence of the habitat conditions like topoclimate, relief or soil on the chemical composition of pollen, which was demonstrated among others by Depciuch et al. (2016) and Zimermann and Kohler (2014). In such planned experiment, the chemical composition is only determined by ‘species’ variable.

### Light and electron microscopy observations

The collected pollen was mounted in glycerogelatin with fuchsine after few days of sampling, which is recommended for observations and measurements under light microscopy. In microscopic samples prepared in such a way, the shape and size of pollen grains are not altered, as in the case of drying or after acetolysis (Reitsma 1969; Clegg et al. 2005). On microscopic slide, the pollen grains were arranged in a way that all the pores were in one plane (polar view). For each species, the size of 50 pollen grains was measured at 400× magnification with the Nicon Eclipse Ci light microscope using NIS software. Karlsdóttir et al. (2007, 2008) defined this size as the distance between two points situated one on the outer layer of exine and the other one on the pora tip.

Scanning electron microscopy was carried out on a TESCAN VEGA 3 SBH instrument equipped with a tungsten cathode. The pollen was deposited on a SEM stub sample holder covered with a carbon patch. The samples were imaged in high vacuum mode at 1 kV accelerating voltage using the SE detector. The pollen samples were observed without being coated with gold or carbon. The pollen grains were collected during rainless season, therefore they were dry and it was not necessary to dry them additionally.

### FTIR measurements

FTIR spectroscopy measurements were taken using a Vertex 70 (Bruker) spectrometer applying the attenuated total reflectance (ATR) technique, with multi-reflective, diamond side disk. The selected infrared radiation was the average IR range (400–4000 cm^−1^). To achieve 4 cm^−1^ spectral resolution, 64 scans were used. For each pollen sample, the same absorption bands corresponding to nucleic acids, proteins, polysaccharides, lipids and water were identified. Each measurement was taken in triplicates. In order to determine the structural changes in pollen grains, the second derivative of the spectra was calculated.

### Data analysis

All spectra were treated with the OPUS software. The analysis of the secondary structure of the proteins was carried out by curve fitting using the GRAMS AI software from Thermo Scientific. The second derivatives were calculated from the ATR–FTIR spectra after smoothing over nine consecutive points. The absorption bands at low wavenumbers were free from features originating from water vapor, as judged from the peaks above 1750 cm^−1^. A straight baseline passing through the ordinate at 1700 and 1610 cm^−1^ was subtracted before the curve fitting. The baseline was again modified by the least-squares curve-fitting software, which allows for a horizontal baseline to be adjusted as an additional parameter to obtain the best fit. The second derivative spectrum was used to determine the initial peak positions for curve fitting, and the peaks were fitted using Gauss functions. The area under the entire band was considered as 100%, and each component after fitting was expressed as a percent fraction.

### Statistical analysis

Firstly, the normal distribution and the homogeneity of variances were checked using Shapiro–Wilk and Brown–Forsyth tests, respectively. The statistical hypothesis about the lack of differences among *Betula* species in respect to the diameter of pollen grains was tested using Kruskal–Wallis test. Dunn’s post hoc test was applied for multiple comparisons. Statistical tests were tested with *α* ≤ 0.05. In order to indicate the groups of the individual species with the highest similarity with respect to the absorbance spectra, hierarchical clustering analysis (HCA) with Euclidean distance and Ward’s algorithms was used. This method was applied for the ranges between 400 and 4000 cm^−1^ (entire spectrum) and 1500 and 1700 cm^−1^ (proteins region), as well as for entire spectra and mean diameter of grains for each species. In latter case, raw data were expressed in different units (μm and cm^−1^); therefore, they were standardized before calculating the Euclidean distance. For HCA, average spectra of each sample were used. Statistical and multidimensional analysis was performed using PAST software.

## Results

### Shape and size of pollen grains

**Fig. 1:**
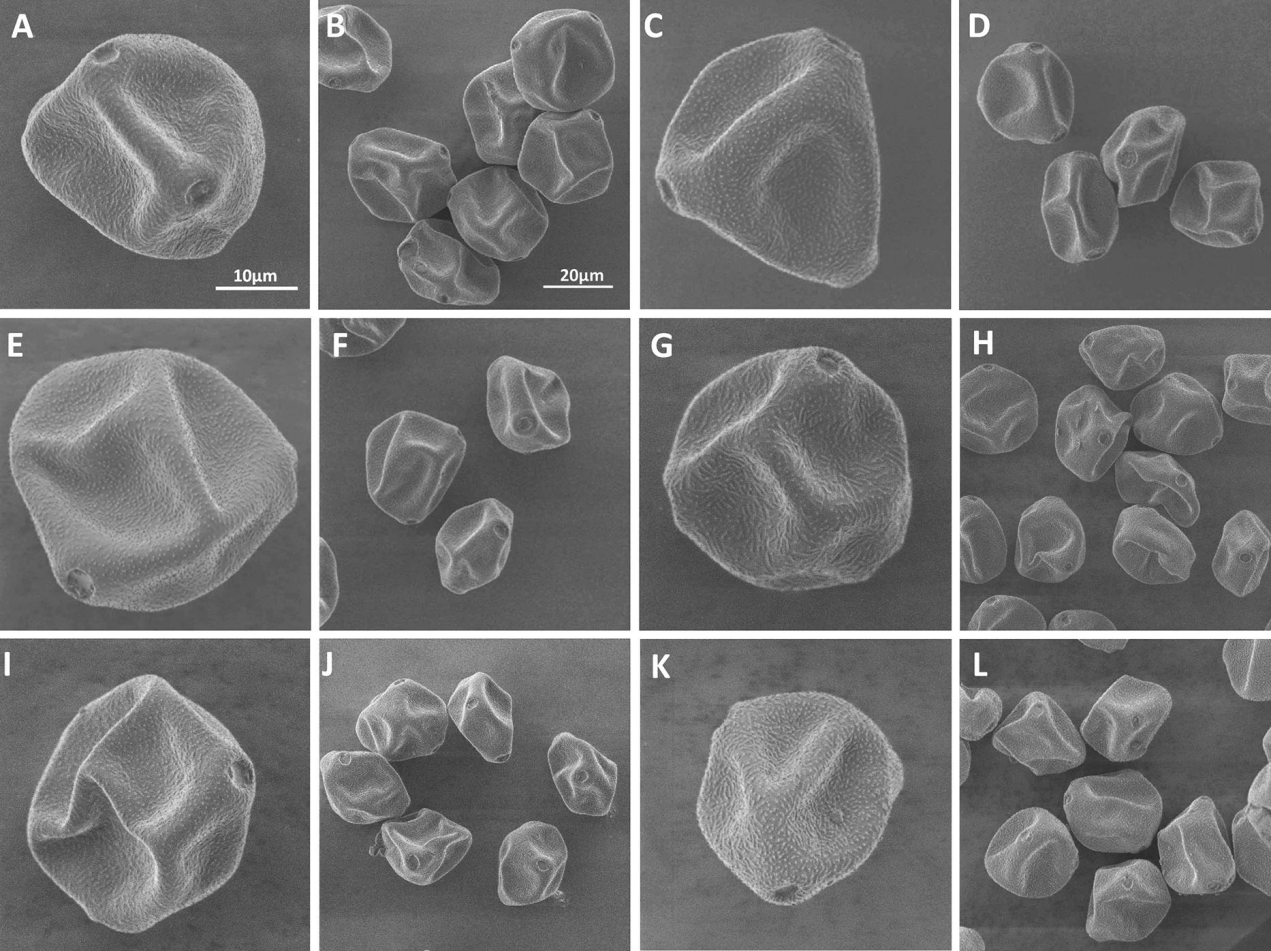
SEM images of different species of birch pollen grains: **a**, **b** *B. utilis Doorenbos*; **c**, **d** *B. utilis dahurica*; **e**, **f** *B. maximowicziana*; **g**, **h** *B. pendula*; **i**, **j** *B. dahurica*; **k**, **l** *B. humilis.* The right-side image is always an overview of pollen. The scale bars correspond to 10 μm in left-side images and 20 μm in right-side images

SEM imaging did not reveal any differences in the shape of the pollen grains of different species (Fig. 1). No distinct differences in exine and pores morphology between the respective samples could be noticed. However, it is clearly visible that the pollen grains have different sizes (Fig. 1). It was confirmed statistically (*H* = 245.446; *p* < 0.000). The *B. utilis Doorenbos* has the largest pollen grains (above 32 μm) followed by *B. maximowicziana* and *B. dahurica*, which pollen grains do not differ significantly from each other. The pollen grains of *B. pubescens*, *B. pendula* and *B. humilis* have similar sizes (from 22.168 to 23.104 μm) and are significantly smaller than the others. The pollen grains of *B. humilis* are smaller than 10 μm from grains of *B. utilis Doorenbos*, and both species are characteristic by great variability (standard deviation values) (Table 1).

**Table 1:** Descriptive statistics of diameter of pollen grains of chosen *Betula* species

*Betula* species	Min (μm)	Max (μm)	Median (μm)	Mean (μm)	SD (μm)
*B. utilis Doorenbos* a	28.15	37.06	32.685	32.614	2.009
*B. dahurica* b	24.02	30.95	27.450	27.329	1.545
*B. maximowicziana* b	24.57	31.73	28.615	28.29	1.518
*B. pendula* c	20.22	25.22	23.220	23.104	1.234
*B. pubescens* c	20.22	26.22	22.975	22.936	1.394
*B. humilis* c	17.64	26.06	22.485	22.168	1.950

*SD* standard deviation; arabic letters denominate groups with differences in size of grains, which was proved by Dunn’s post hoc test

### Molecular composition

**Fig. 2:**
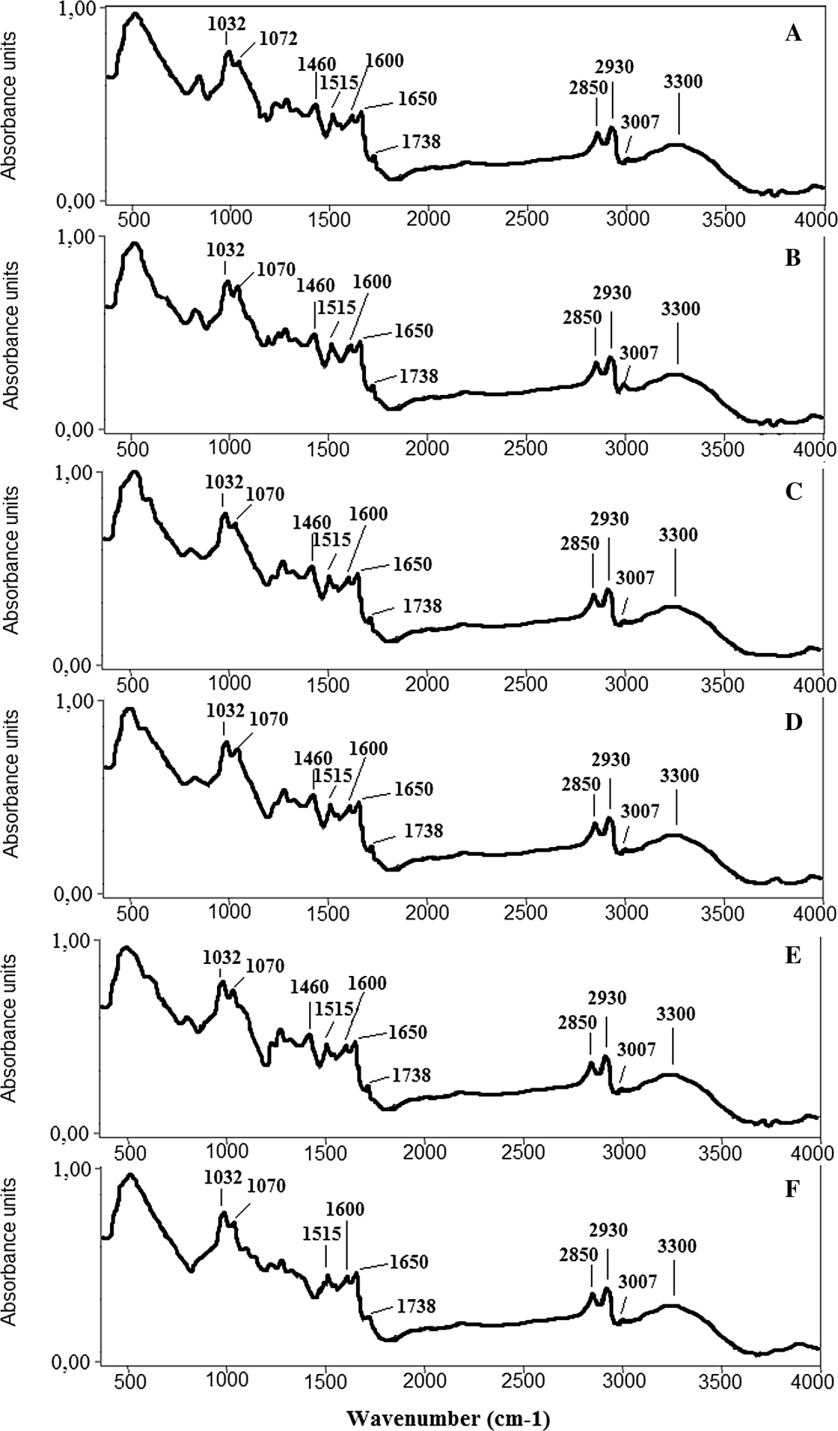
Offset of average FTIR spectra of *Betula* pollen: **a** *B. utilis Doorenbos*, **b** *B. dahurica*, **c** *B. maximowicziana*, **d** *B. pendula*, **e** *B. pubescens* and **f** *B. humilis*

Figure 2 shows the offset of the FTIR spectra indicating specific bonds for pollen from each site. Main absorption bands and their assignments are presented in Table 2. For each pollen sample, the same absorption bands corresponding to polysaccharides, proteins, lipids and water were identified (Table 2, Fig. 2). The low wave number region of the FTIR spectrum originates from the chemical bonds of polysaccharides (1032, 1000–1075 cm^−1^) and proteins (1460, 1515–1570, 1600, 1650 cm^−1^). The peak at 1738 cm^−1^ corresponds to stretching vibrations of the C=O group, which evidences the presence of lipids. Vibrations observed in the FTIR spectrum at 2850 and 2930 cm^−1^ wave numbers originate from CH_2_ and CH_3_ vibrations of lipids, proteins and carbohydrates. The peaks at 3007 and 3300 cm^−1^ correspond to C=C and O–H vibrations (Rubio-Diaz et al. 2010; Mohani et al. 2014; Rather et al. 2014).

**Table 2:** Values of wave numbers with the corresponding vibrations present in pollen of six *Betula* species

No.	Wavenumber (cm^−1^)	Vibrations
1	1032	Stretching vibrations of C–O–C group
2	1000–1075	C–O stretching
3	1460^a^	CH_2_ stretching
4	1515–1570	–CNH (amide II)
5	1600	C=O stretching (proteins)
6	1650	N–H_2_ bending (amide I)
7	1738	C=O stretching (lipids)
8	2850	CH_2_ stretching (lipids)
9	2930	CH_3_ stretching (lipids)
10	3007	C=C
11	3300	Deformation vibrations of O–H (water)

^a^Missing in spectrum of *B. humilis*

FTIR spectra (Fig. 2) show that in the five pollen samples (*B. utilis Doorenbos*, *B. dahurica*, *B. maximowicziana, B. pendula*, *B. pubescens*), the same functional groups were observed, while the spectrum of *B. humilis* pollen (Fig. 2f) does not show the presence of vibrations at wave number 1460 cm^−1^, which corresponds to the stretching vibration of the CH_2_ group derived from proteins and lipids. However, the values of maximum absorbance of individual peaks differ from each other in all six pollen samples, which means that there are quantitative differences among the six pollen species in terms of chemical composition.

To obtain structural information from the samples, the second derivative from the respective peaks is calculated.

**Fig. 3:**
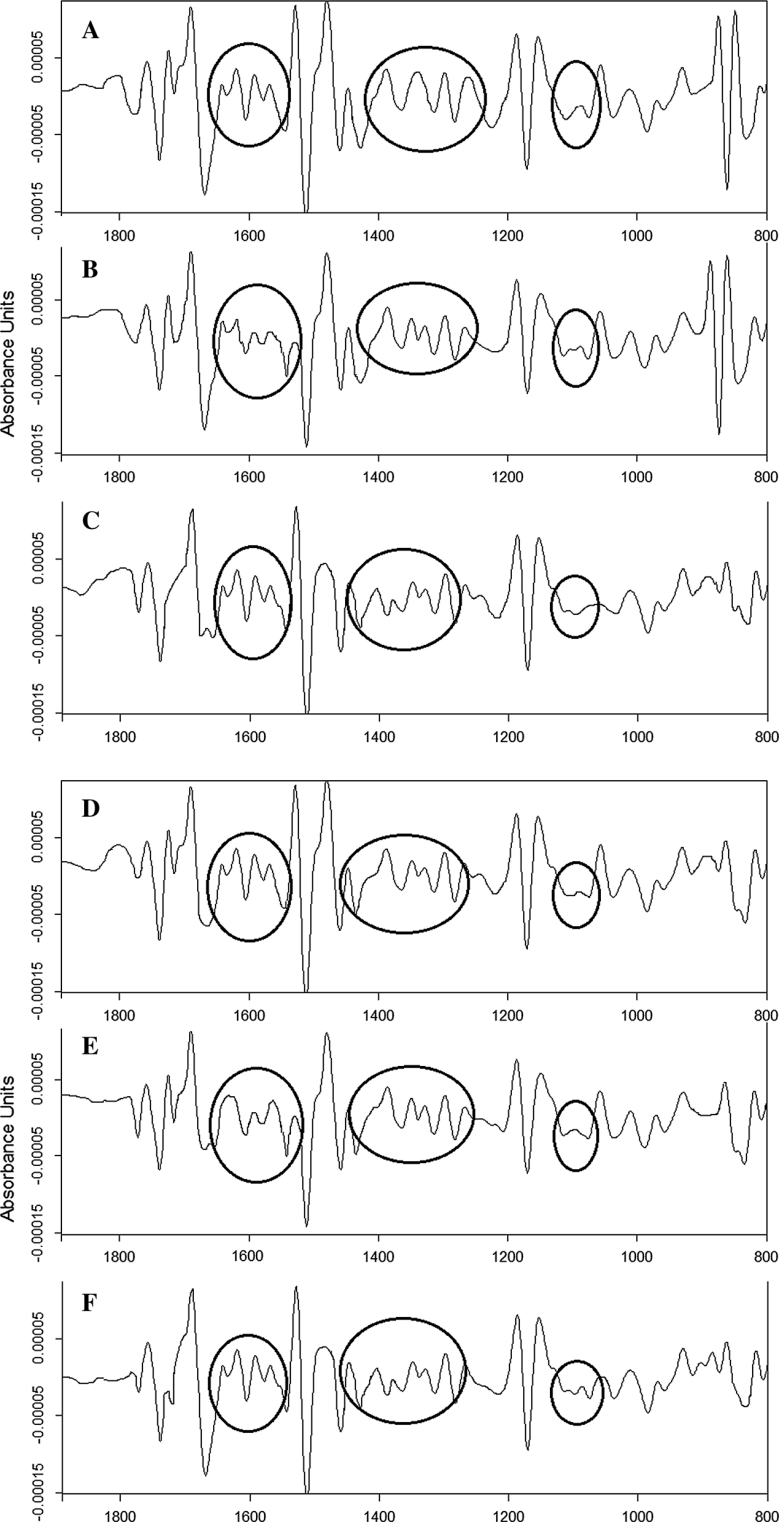
Second derivative of the FTIR spectra of *Betula* pollen: **a** *B. utilis Doorenbos*, **b** *B. dahurica*, **c** *B. maximowicziana*, **d** *B. pendula*, **e** *B. pubescens* and **f** *B. humilis*

**Fig. 4:**
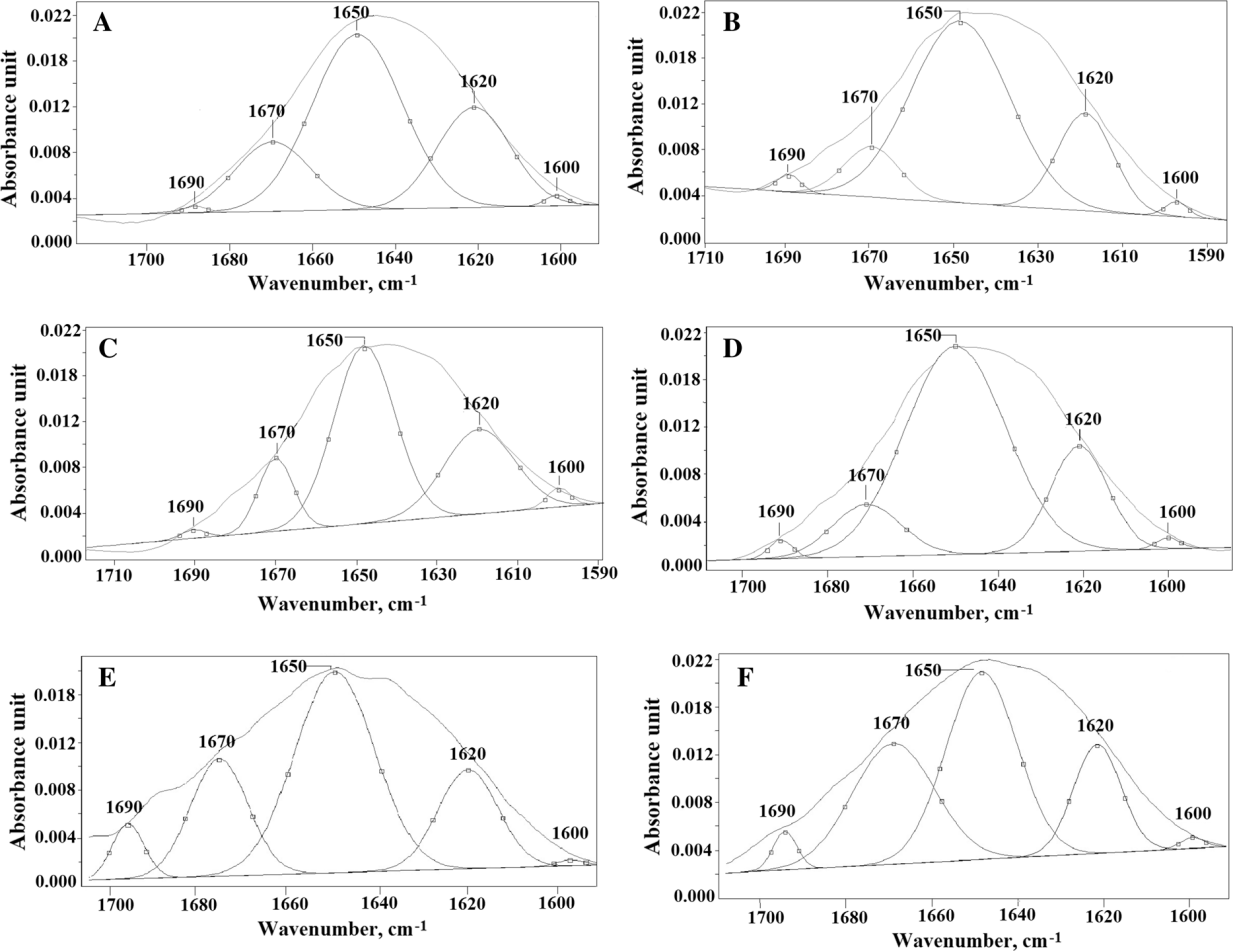
Curve-fitting analysis of the amide I profile of *Betula* pollen: **a** *B. utilis Doorenbos*, **b** *B. dahurica*, **c** *B. maximowicziana,* **d** *B. pendula*, **e** *B. pubescens* and **f** *B. humilis*

From the calculated second derivative of the FTIR spectra (Fig. 3), it can be noted that the most visible structural differences between each species were observed in the protein (1300–1700 cm^−1^) and carbohydrates and lipids (1000–1250 cm^−1^) regions. Moreover, it should be mentioned that in the identification of pollen by FTIR technique, the region in the range between 800 and 1800 cm^−1^ is the so-called fingerprint region. In this area, the highest differences between species were noticed. Furthermore, significant variations of the second derivative of the FTIR spectra in the protein region may indicate structural changes in proteins fraction between pollen samples. In order to obtain information about the type of secondary structural changes between pollen samples of α-helix and β-harmonica of proteins (Dogan et al. 2007; Misra et al. 2015; Pandey et al. 2010; Mauerer and Lee 2006; Maury et al. 2005), thus curve-fitting analysis of amide I profile was performed (Fig. 4).

Table 3 shows the percentage content and the vibration type of the protein’s secondary structures for the six analyzed pollen samples. As the structural changes in proteins between pollen samples are visible only in amide I bonds (1600–1700 cm^−1^), the curve-fitting analysis was performed only for this region (Fig. 4). All peaks corresponding to the secondary structure of proteins are visible in each of the six pollen spectra. The peaks at 1600 and 1620 cm^−1^ correspond to β-sheet vibrations. The maximum absorbance at wave number 1650 cm^−1^ originates from α-helix vibrations. Vibrations observed in the curve-fitting analysis of the amide I profile at 1670 and 1690 cm^−1^ correspond to the β-turn (Table 4).

**Table 3:** Curve-fitting analysis of protein’s secondary structures of *Betula* pollen: A—*B. utilis Doorenbos*, B—*B. dahurica*, C—*B. maximowicziana,* D—*B. pendula*, E—*B. pubescens*, F—*B. humilis*

No.	Wave number (cm^−1^)	Values (%)					
		A	B	C	D	E	F
1	1600	0.8	1.5	2.3	0.4	1.1	0.8
2	1620	25.5	20.2	30.4	10.0	20.8	19.9
3	1650	54.6	66.5	53.7	49.8	40.3	43.9
4	1670	18.8	10.40	12.5	35.9	18.3	32.5
5	1690	0.1	1.45	1.1	4.0	19.5	2.9

**Table 4:** Wave numbers with the corresponding vibrations of protein secondary structures. Reproduced with permission from Dogan et al. (2007), Misra et al. (2015), Pandey et al. (2010), Mauerer and Lee (2006), Maury et al. (2005)

Wave number (cm^−1^)	Vibrations
1600	β-Sheet
1620	β-Sheet
1650	α-Helisa
1670	β-Turn
1690	β-Turn

**Fig. 5:**
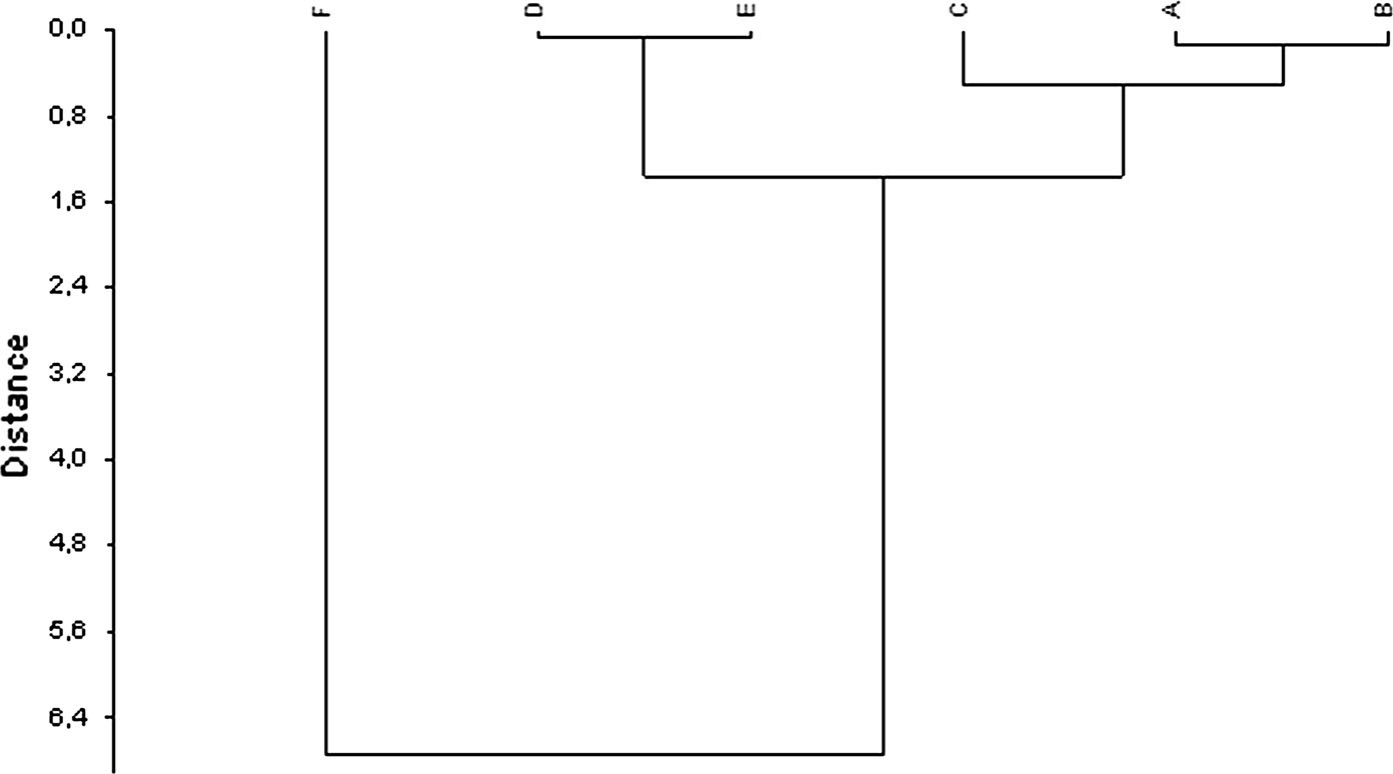
Homogeneous groups according to the highest similarity of entire FTIR spectra of *Betula* pollen: **a** *B. utilis Doorenbos*, **b** *B. dahurica*, **c** *B. maximowicziana,* **d** *B. pendula*, **e** *B. pubescens* and **f** *B. humilis*

The results of HCA showed which *Betula* species are similar to each other taking into account the data obtained from FTIR. The analysis of the dendrogram revealed two groups of species with distinctly different spectra, as well as one outlying specie (Fig. 5).

**Fig. 6:**
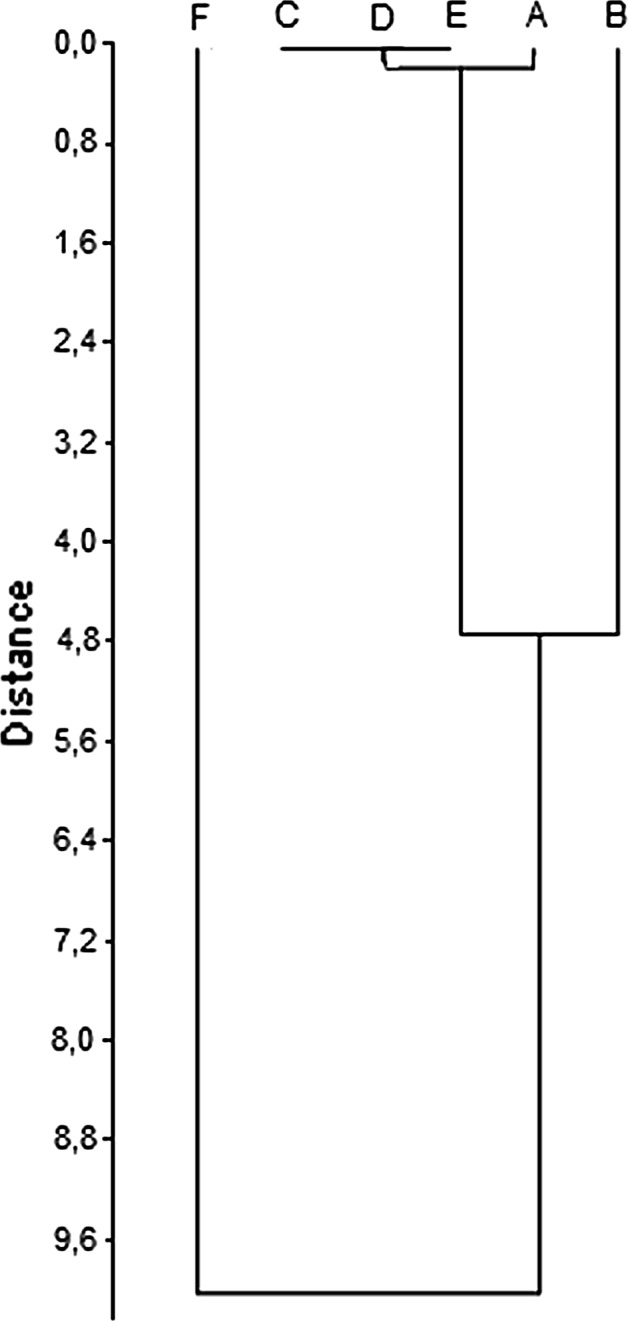
Homogeneous groups according to the highest similarity of FTIR spectra of *Betula* pollen in the range between 1500 and 1700 cm^−1^ **a** *B. utilis Doorenbos*, **b** *B. dahurica*, **c** *B. maximowicziana*, **d** *B. pendula*, **e** *B. pubescens* and **f** *B. humilis*

According to the chemical composition, cluster analysis shows that three homogeneous groups can be distinguished. The first one includes *B. pendula* (D) and *B. pubescens* (E), which show the highest similarities. The second group consists of *B. utilis Doorenbos* (A), *B. dahurica* (B) and of *B. maximowicziana* (C). *B. humilis* (F) clearly stands out from all other species (Fig. 5). Also HCA of the protein region (FTIR spectrum between 1500 and 1700 cm^−1^) shows that *B. humilis* (F) is different from all other species (Fig. 6). Protein compositions of *B. pendula* (D), *B. pubescens* (E), *B. maximowicziana* (C) and *B. utilis Doorenbos* (A) are very similar to each other. *B. dahurica* (B) is slightly different from this last group.

**Fig. 7:**
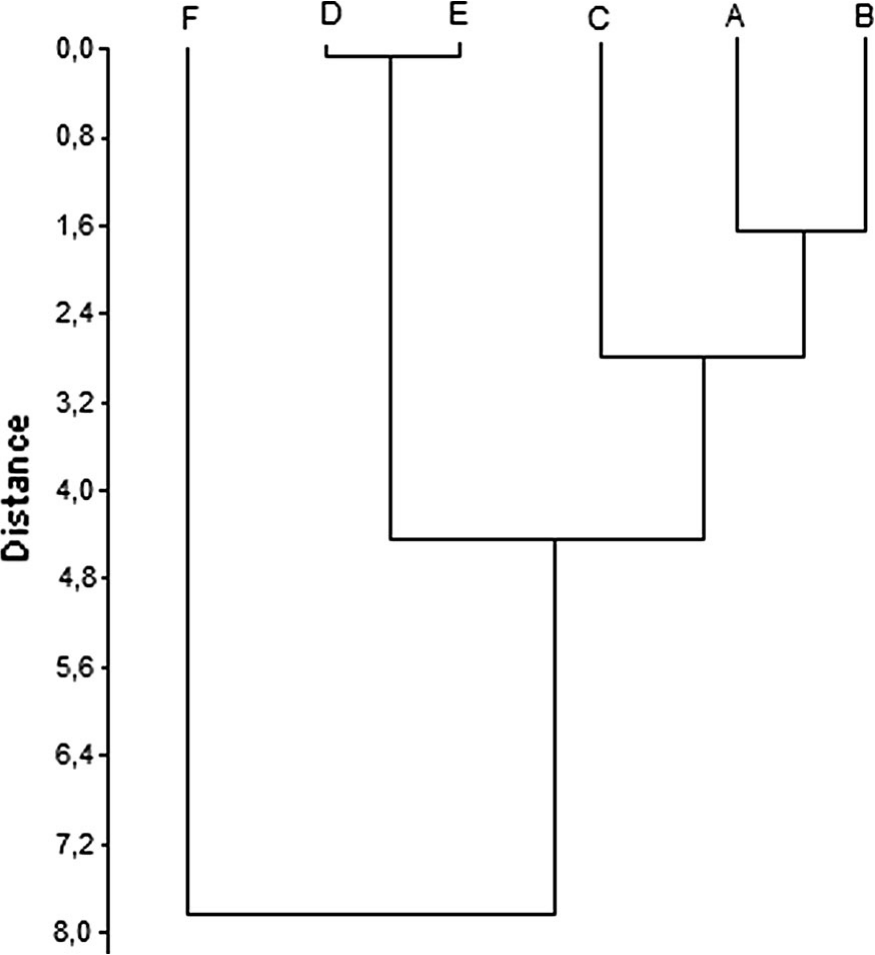
Homogeneous groups according to the highest similarity of entire FTIR spectra of spectra of *Betula* pollen and pollen grains diameter: **a** *B. utilis Doorenbos*, **b** *B. dahurica*, **c** *B. maximowicziana,* **d** *B. pendula*, **e** *B. pubescens* and **f** *B. humilis*

In addition, HCA was performed on Euclidean distances between pollen diameter values to verify whether the biometric approach may be useful to better differentiate between *Betula* species. The results presented in Fig. 7 are really similar to the ones shown in Fig. 5, and only small differences concerning the value of Euclidean distances were observed. The similarities between *B. maximowicziana* (C), *B. utilis Doorenbos* (A) and *B. dahurica* (B) are weaker in HCA performed on pollen diameter than in HCA performed on results obtained for FTIR measurements.

## Discussion

Birch pollen is a frequent object of aerobiological and paleopalynological studies. It contains one of the strongest pollen allergens, and it is also one of the most important components of quaternary pollen spectra. Some *Betula* species are indicators of different types of vegetation, and based on the presence of their pollen grains in the sediments, paleoclimate can be inferred (Blackmore et al. 2003). Therefore, the ability to distinguish pollen grains to species level is of great importance.

The analysis indicated that *B. utilis Doorenbos* pollen is easily recognizable from all the others, as it has significantly larger dimensions. *B. pubescens*, *B. pendula* and *B. humilis* have pollen grains of similar size, even if the results obtained in the present study for two first species clearly differ from the ones reported by other authors (Mäkelä 1996; Karlsdóttir et al. 2007). According to Mäkelä (1996), pollen grains of *B. pendula* (20–25 μm in average) are generally smaller than those of *B. pubescens* (22–26 μm in average). They have stressed that *B. pubescens* is a very variable species and ‘small’ grains of *B. pubescens* are not so rare. That is what makes distinguishing between these two species problematic.

The taxonomy of *Betula* genus is still challenging. One of the reasons is its ability to create hybrids. The differentiation among species or varieties on the basis of morphology is often difficult, because of phenotypic plasticity and ecotype variation; in this respect, biochemical and genetic methods could be helpful (Keinänen et al. 1999; Järvinen et al. 2004).

There is no literature about using FTIR to distinguish more than two *Betula* species. However, Atkinson et al. (1997) used IR spectroscopy for discriminating between *B. pendula*, *B. pubescens*, as well as their hybrids. They analyzed leaves, including the petiole, showing that it is possible to define discriminant functions based on selected spectral wavelengths, able to perform a correct classification in most of cases. However, there is a risk that by increasing the number of spectral wavelengths the discriminant functions will not perform well on other data. Dieterich (1963), Nokes (1979) and Atkinson et al. (1997) showed that a hybrid is more similar to the higher ploidy level parent (e.g., *B. pubescens*, 2*n* = 56) than to the lower ploidy level parent (*B. pendula*, 2*n* = 28). Moreover, depending on the plant part, from which the sample was taken, the FTIR spectra are different (Atkinson et al. 1997). They obtained different FTIR spectra from different plant parts, e.g., leaf, stem, pollen, root. Therefore, to perform classification of species using FTIR spectroscopy, the samples collected from the same plant part must be measured. Therefore, in the present study only pollen grains were analyzed by FTIR. Moreover, FTIR spectra of biological systems are very complex, being due to the overlapping absorption of the main biomolecules. For this reason, it is necessary to apply an appropriate multivariate analysis, able to process very high-dimensional data, to pull out significant and non-redundant information contained in the spectra (Ami et al. 2013; Lõoke et al. 2011). Moreover, *Betula* species, including these analyzed in the present study, vary in origin, habitat requirements and form, as well as in the ability to create hybrids (Atkinson 1992; Järvinen et al. 2004). In Poland, the most common species is *B. pendula* (silver birch), which is native for Europe and Northern Asia, as well as in Morocco. *B. pubescens* (downy birch), the second native birch species in Poland occurs also in Greenland, Europe to Russian Far East and N. Iran (Atkinson 1992). Both species belong to subgenus *Betula* (Järvinen et al. 2004). Although their habitat requirements are different, they have the tendency to create hybrids (Atkinson 1992; Järvinen et al. 2004). On the base of cluster analysis (Fig. 5), we can suppose that the molecular compositions of pollen of these species are very similar. Species belonging to the second group defined by the HCA analysis are not native for Poland or Europe, but grow well, when planted in suitable conditions. These are *B. dahurica* (Dahurian birch) native from China, Japan and Korea, *B. maximowicziana* (monarch birch) originating from Japan, as well as *B. utilis Doorenbos*, usually an ornamental tree in urban parks and in large gardens. HCA showed that *B. humilis*, which belongs to *Chamaebetula* subgenus (Järvinen et al. 2004), stands out from other species. It is native to subarctic and subalpine areas in Eurasia and is a boreal relic for Poland, where it occurs only in few sites and is protected by law (endangered category). It occurs mainly in peatlands and wet meadows as low and strongly branched shrub (Załuski et al. 2001).

Spectroscopy is an effective method of pollen identification, especially when the species are not phylogenetically related to each other. In the case of a strong relationship, the spectra are similar to each other (Zimmermann 2018). In 2017, we examined the chemical composition of hazel pollen (Depciuch et al. 2017), a species closely related to birches (Järvinen et al. 2004). The mere visual comparison of the spectra reveals clear differences, which indicate that the chemical composition of hazel pollen distinctly differs from the chemical composition of pollen of all birch species.

Summarizing, spectroscopy measurements indicate that the molecular composition of *B. humilis* is clearly distinct from the other species, but the size of the pollen grains does not statistically differ from the size of *B. pendula* and *B. pubescens*. On the other hand, the diameter of *B. utilis Doorenbos* pollen grains is the largest, but its FTIR spectrum is similar to *B. maximowicziana* and *B. dahurica* spectra, whose pollen grains are significantly smaller.

## Conclusions

Distinguishing birch pollen to species level is important in paleoecology and studies dealing with the content of allergenic pollen in air. In the present work, an attempt to distinguish between birch pollen of six species by comparing their chemical composition was undertaken. The obtained results show that FTIR spectroscopy is a fast reliable technique for this purpose. *B. humilis* is the most outstanding species from the six analyzed samples in terms of chemical composition. It is weakly related to other species; moreover, it is distinguished by specific habitat requirements, the area of origin and a small area of occurrence. It seems that in the case of birches, the chemical composition is related to these characteristics. In this perspective, spectroscopic methods may give an interesting outlook in research concerning ecology or origin of species, and molecular taxonomy. The presented biometric method allowed to distinguish three groups of species, which differ in terms of pollen grains diameters, yet its taxonomic significance is weaker because it relies only on one feature (the size), while spectroscopy analyzes the content of many chemical compounds. The results show that the pollen collected from the flower can be easily distinguished by means of spectroscopy, and thus, this method has the potential to be used in continuous monitoring of aeroallergens. Biometric analysis could be used as a preliminary tool, while spectroscopy would be applied as a more specific technique. At the moment, however, there are no methodological solutions allowing to collect samples from air, which could be subjected to spectroscopy.
